# Differential Gene Expression across Breed and Sex in Commercial Pigs Administered Fenbendazole and Flunixin Meglumine

**DOI:** 10.1371/journal.pone.0137830

**Published:** 2015-09-14

**Authors:** Jeremy T. Howard, Audrey T. O’Nan, Christian Maltecca, Ronald E. Baynes, Melissa S. Ashwell

**Affiliations:** 1 Department of Animal Science, College of Agriculture and Life Sciences, North Carolina State University, Raleigh, NC, United States of America; 2 Department of Population Health and Pathobiology, Center for Chemical Toxicology and Research Pharmacokinetics, College of Veterinary Medicine, North Carolina State University, Raleigh, North Carolina, United States of America; University of Florida, UNITED STATES

## Abstract

Characterizing the variability in transcript levels across breeds and sex in swine for genes that play a role in drug metabolism may shed light on breed and sex differences in drug metabolism. The objective of the study is to determine if there is heterogeneity between swine breeds and sex in transcript levels for genes previously shown to play a role in drug metabolism for animals administered flunixin meglumine or fenbendazole. Crossbred nursery female and castrated male pigs (n = 169) spread across 5 groups were utilized. Sires (n = 15) of the pigs were purebred Duroc, Landrace, Yorkshire or Hampshire boars mated to a common sow population. Animals were randomly placed into the following treatments: no drug (control), flunixin meglumine, or fenbendazole. One hour after the second dosing, animals were sacrificed and liver samples collected. Quantitative Real-Time PCR was used to measure liver gene expression of the following genes: *SULT1A1*, *ABCB1*, *CYP1A2*, *CYP2E1*, *CYP3A22* and *CYP3A29*. The control animals were used to investigate baseline transcript level differences across breed and sex. Post drug administration transcript differences across breed and sex were investigated by comparing animals administered the drug to the controls. Contrasts to determine fold change were constructed from a model that included fixed and random effects within each drug. Significant (P-value <0.007) basal transcript differences were found across breeds for *SULT1A1*, *CYP3A29* and *CYP3A22*. Across drugs, significant (P-value <0.0038) transcript differences existed between animals given a drug and controls across breeds and sex for *ABCB1*, *PS* and *CYP1A2*. Significant (P <0.0038) transcript differences across breeds were found for *CYP2E1* and *SULT1A1* for flunixin meglumine and fenbendazole, respectively. The current analysis found transcript level differences across swine breeds and sex for multiple genes, which provides greater insight into the relationship between flunixin meglumine and fenbendazole and known drug metabolizing genes.

## Introduction

Drug use in livestock has received increased attention due to welfare concerns and food safety but a limited amount of work has been done on any potential genetic variation in drug metabolism that exists within livestock species and its impact on withdrawal times in commercial swine production. Recent work by Howard et al. [1] has shown that differences in pharmacokinetic parameters exist across four major swine breeds (i.e. Duroc, Hamphsire, Yorkshire and Landrace) and sex. This is unsurprising due to the vast body of literature in humans that has identified individual and group genetic variation in drug metabolism and response [2–6]. The pig has recently become increasing relevant as an animal model for biomedical research, mainly due to the fact that the anatomy, genetics and physiology reflect human biology more closely than classic animal models (i.e. fruit fly, zebrafish and rodents) [7]. Furthermore, differences in transcript levels have been shown to exist in commercial pigs and special breeds such as the Göttinger minipig and the Yucatan micropig [8–9]. Fink-Gremmels [10] examined liver cytochrome P450 enzymatic activity levels and found that conventional pigs have activity levels similar to those found in human livers while miniature pigs have much higher activity levels. Many drugs used to treat livestock are similar to those used in humans, which allows for knowledge to be garnered that will impact both the swine industry and human drug development [11]. Additionally, within the past decade the identification of a large number of single nucleotide polymorphisms (SNP), the sequencing of the swine genome, and a large list of quantitative trait loci across multiple economically important traits have been identified [12]. Therefore, further insight into drug-metabolizing enzyme biology using the commercial pig as a model provides great opportunity for the livestock industry and as a research tool in human medicine to move closer to the ultimate goal of giving the right drug at the right dose to the right patient at the right time.

An extremely limited amount of work in swine has been conducted on relating differences based on pharmacokinetic parameters to differences at the gene expression level, although this has been well documented in humans [13–14]. The cytochrome P450 family is a large group of monooxygenase enzymes expressed in liver that are major players in drug metabolism, accounting for ~75% of all drug metabolizing enzymes [15]. Despite the highly conserved function of the CYP450 family in most mammals, significant species differences exist at the level of expression and substrate specificity [10, 16]. It has been shown in minipigs and micropigs that females have higher enzymatic activity for *Cytochrome P450 1A2* (*CYP1A2*) and *Cytochrome P450 2E1* (*CYP2E1*) in comparison to males [10]. The *Cytochrome P450 3A4* (*CYP3A4*) gene in adult humans is the most important drug-metabolizing enzyme due to its prominent expression in liver and the gut and has been shown to account for 66% to 88% of the inter-individual variation in drug response [17]. The swine homologue to *CYP3A4* gene is *Cytochrome P450 3A22* (*CYP3A22*) and *Cytochrome P450 3A29* (*CYP3A29*) and both genes have been shown to be highly expressed in the liver of Bama miniature pigs [18].

Genes outside of the cytochrome P450 family, including The ATP-Binding Cassette Sub-Family B (*ABCB1*) and *Sulfotransferase Family*, *Cytosolic*, *1A*, *Phenol-Preferring*, *Member 1* (*SULT1A1*) have also been shown to play a role in drug metabolism. The *ABCB1* gene is thought to play an important role in removing toxic substances or metabolites from cells and multiple SNP in this gene have been shown to impact drug clearance in humans [19]. Furthermore, genetic variability within the *ABCB1* gene has been characterized in domestic animal species [20] and has been shown to be segregating in multiple dog breeds [21]. Lastly, genetic polymorphisms have been reported in the *Sulfotransferase Family*, *Cytosolic*, *1A*, *Phenol-Preferring*, *Member 1* (*SULT1A1*) gene in swine, which resulted in a significant decrease in sulfation activity [22]. Sulfation is one of the major conjugation reactions involved in the metabolism of drugs and xenobiotic compounds and a great deal of research on this particular conjugation reaction has been conducted in humans [23].

Fenbendazole and flunixin meglumine are drugs used across a variety of livestock species and pharmacokinetic differences across breed and sex have been shown to exist when these drugs are administered [1], although they are not cleared for use in humans. Fenbendazole is a widely used antihelmintic drug due to its broad-spectrum activity and is often administered as a feed additive. It undergoes CYP450-mediated oxidation and conjugation with glucoronide and sulfate [24]. Flunixin meglumine is used for the control of pyrexia associated with swine respiratory disease. Flunixin meglumine inhibits the cyclo-oxygenase enzyme, thereby decreasing prostaglandin synthesis and is hydroxylated in the liver to 5-hydroxy flunixin [25]. The objective of the study was to determine if gene expression differences exist across breed and sex for animals given flunixin meglumine or fenbendazole for genes previously shown to play a role in drug metabolism, including *CYP1A2*, *CYP2E1*, *CYP3A22*, *CYP3A29*, *ABCB1* and *SULT1A1*.

## Materials and Methods

### Animals and experimental design

This study was approved by the NCSU Institutional Animal Care and Use Committee (IACUC). The experimental design was similar to the one outlined by Howard et al. [1]. Briefly, crossbred nursery female and castrated male pigs (n = 181) with an average (± SD) weight (kg) of 30.59 (±5.82) were utilized. The animals were housed in individual pens. Because only 32 pens were available, the animals were spread across five groups. The number of animals within each group was 31, 29, 25, 26, and 30 for groups from 1 to 5, respectively. The first group occurred on October 19, 2013 and the last group occurred on January 12, 2015. All animals were fed an *ad libitum* standard commercial corn and soybean meal based diet throughout the study.

The sires (n = 15) of the pigs were registered National Swine Registry boars mated to a common sow (Yorkshire X Landrace; n = 34) population at the North Carolina State University Swine Education Unit. Individual sows were not used for more than one mating and the average (max-min) number of pigs for a sire and dam used in the study was 12.7 (31–6) and 5.6 (14–2), respectively. The boars utilized were purebred Duroc (n = 4), Hampshire (n = 3), Landrace (n = 5) and Yorkshire (n = 3). Within each group animals were randomly placed into the following treatment groups: no drug (control), flunixin meglumine administered, or fenbendazole administered. The number of individuals within a particular breed and sex varied across groups due to conception issues, therefore some degree of unbalance did exist, but it was minimized as much as possible.

### Drug administration and tissue collection

Prior to the start of the drug administration and tissue collection, pigs were moved to individual pens and jugular catheters were inserted. After a recovery period of 3 days, drugs were administered by intravenous (IV) administration. Intravenous administration could potentially fail to detect differences in drug uptake from extravascular administration across breed and potential differences may not extrapolate to extravascular administration, however the IV route was selected to remove inter- and intra-individual variability often observed with extravascular routes of administration [24, 26]; thereby focusing the variability on blood clearance mechanisms within the pig. Single IV injections of all drugs were at fractions of the estimated bio-available dose to reduce the chance of saturating metabolic enzymes.

Preparation of fenbendazole (0.4 grams) (Sigma-Aldrich, Co., St. Louis, MO, USA) was done by dissolving the drug in 20 mL DMSO (dimethyl sulfoxide) and 80 mL PG (propylene glycol or 1,2-propanediol) and used to administer an average single intravenous dose of 1 mg/kg fenbendazole to each animal. Banamine® Injectable Solution (50 mg/mL; each mL contains 50 mg flunixin as the meglumine salt) (Merck Animal Health, Summit, NJ) was used to administer an average single intravenous dose of 3 mg/kg flunixin meglumine to each animal. An initial dose was given in order to collect blood samples across a 48-hr period, although this data was not utilized in the study. Previous work by Howard et al. [1] has shown that negligible amounts of the drug and metabolite remained in the blood plasma after 48 hours. Then a second dose was given and, one hour after drug administration, animals were sacrificed via captive bolt and a liver sample from the same lobe in all animals was collected in RNAlater (Qiagen, Valencia, CA) following the manufacturers’ protocol. A liver sample collection of one hour after drug administration was chosen based on a previous study by Howard et al. [1] that found high levels of the metabolite and moderate levels of the drug in the blood plasma across both drugs. Control animals were sacrificed on the same day as the dosed pigs and a similar liver sample was collected as described above.

### Quantitative real-time PCR (qPCR)

Total RNA from approximately 30mg of liver was isolated using the RNeasy Plus Mini kit (Qiagen, Valencia, CA) following the manufacturers’ protocol. Integrity and purity of the RNA were measured and quantitated on a Nanodrop-1000 spectrophotometer (Thermo Scientific, Wilmington, DE) and visualized on a 1.2% denatured agarose gel. cDNA was synthesized from 500ng of total RNA using the iScript Reverse Transcription Supermix (Bio-Rad, Hercules, CA). Primer pairs for quantitative real-time PCR (qPCR) were selected for the following target genes: *CYP1A2*, *CYP2E1*, *CYP3A22*, *CYP3A29*, *ABCB1* and *SULT1A1*. Primer pairs for four housekeeping genes were also selected. These were *Beta-actin* (*ACTB*), *hypoxanthine phosphoribosyltransferase 1* (*HPRT1*), *ribosomal protein L4* (*RPL4*) and *TATA box binding protein* (*TBP*). These housekeeping genes were selected based on previous work by Fry et al. [27], where it was found that these genes were the most stable in the pig liver. For genes without previously published primer sequences, primers were designed with NCBI Primer-BLAST software (http://www.ncbi.nlm.nih.gov/tools/primer-blast) for compatibility with SYBR Green I Master Mix. The primers were designed (S1 Table) to cross an intron-exon boundary when possible. qPCR was performed in a 20μL reaction, consisting of 1μL of 1:20 diluted cDNA, 400nM of forward and reverse primers, and iTaq™ Universal SYBR® Green Supermix (Bio-Rad, Hercules, CA). A two-step amplification protocol was performed in an iCycler IQ Real-Time PCR Detection System (Bio-Rad, Hercules, CA) with the following steps: denaturation with one cycle at 95°C for 3 minutes followed by 35 cycles of 15 sec at 95°C for denaturation, 45 sec at 60°C for annealing, extension, and data collection. The gene target specificity of the reactions was evaluated with a melt curve generated at the end of the PCR amplification cycle. Melt curve analysis was 80 cycles starting at 55°C and increasing 0.5°C every cycle, with a dwell time of 15sec. Samples were amplified in duplicate, and reaction efficiency for each primer set was assessed using standard curves via a dilution series using iCycler iQ Software. Duplicate samples that differed by greater than one were removed and not included in the analysis for a given animal. No template controls were run on every plate. One cDNA product from each primer pair was sequenced to verify that the PCR product corresponded to the intended gene.

### Statistical analysis

Three linear mixed models that have a similar framework as Steibel et al. [28] were used to analyze the data in order to determine the basal expression differences across breed and sex and to determine the effect of drug administration on changes in gene expression and whether this varied across breed and sex. A target gene along with the four housekeeping genes was analyzed within each drug, which resulted in 6 separate analyses within each drug across all three models. Prior to the final analysis across all models, conditional studentized residuals were checked for normality and potential outliers. A heterogeneous residual variance by gene was investigated for all models and the Akaike information criterion (AIC) value did not improve therefore a homogenous residual variance across genes was utilized. The number of individuals used in the final analysis by breed and sex for animals given flunixen meglumine or fenbendazole and the controls are outlined in Table 1 and a more thorough list by group is given in S2 Table. Due to the removal of duplicate samples that differed by greater than one cycle threshold during amplification, the total number of data points by gene may differ. Furthermore, the complete dataset used in the analysis is included as a S1 File.

**Table 1:** The number of animals by breed and sex for flunixin meglumine, fenbendazole and the control used in the final analysis across target and housekeeping genes^1^.

		Breed				Sex	
Treatment	Gene	Duroc	Yorkshire	Hampshire	Landrace	Female	Male
flunixin meglumine	*ABCB1*	23	13	17	14	32	35
flunixin meglumine	*SULT1A1*	22	14	19	15	32	38
flunixin meglumine	*CYP1A2*	23	13	19	15	32	38
flunixin meglumine	*CYP2E1*	19	12	18	14	29	34
flunixin meglumine	*CYP3A22*	21	14	19	14	32	36
flunixin meglumine	*CYP3A29*	22	14	19	15	31	38
flunixin meglumine	*ACTB*	22	14	19	15	32	38
flunixin meglumine	*HPRT*	23	13	19	15	32	38
flunixin meglumine	*RPL4*	22	13	19	15	30	39
flunixin meglumine	*TBP*	20	13	19	15	30	37
fenbendazole	*ABCB1*	16	14	14	11	26	29
fenbendazole	*SULT1A1*	17	15	16	11	27	32
fenbendazole	*CYP1A2*	16	15	16	10	27	30
fenbendazole	*CYP2E1*	16	15	15	10	26	30
fenbendazole	*CYP3A22*	15	14	14	9	23	29
fenbendazole	*CYP3A29*	17	13	16	10	25	31
fenbendazole	*ACTB*	17	14	16	11	26	32
fenbendazole	*HPRT*	16	14	15	11	25	31
fenbendazole	*RPL4*	17	15	16	10	26	32
fenbendazole	*TBP*	16	15	16	10	25	32
control	*ABCB1*	10	4	11	12	19	18
control	*SULT1A1*	10	6	11	10	18	19
control	*CYP1A2*	10	5	11	11	18	19
control	*CYP2E1*	9	5	12	11	19	18
control	*CYP3A22*	9	5	11	10	18	17
control	*CYP3A29*	10	5	11	12	20	18
control	*ACTB*	10	5	12	12	20	19
control	*HPRT*	10	4	11	11	18	18
control	*RPL4*	9	5	12	12	19	19
control	*TBP*	10	6	12	12	20	20

^1^ Abbreviations: *ATP-Binding Cassette Sub-Family B* (*ABCB1*); *Sulfotransferase Family*, *Cytosolic*, *1A*, *Phenol-Preferring*, *Member 1* (*SULT1A1*); *Cytochrome P450 1A2* (*CYP1A2*); *Cytochrome P450 1E2* (*CYP2E1*); *Cytochrome P450 3A22* (*CYP3A22*); *Cytochrome P450 3A29* (*CYP3A29*); *Beta-actin* (*ACTB*); *hypoxanthine phosphoribosyltransferase 1* (*HPRT1*); *ribosomal protein L4* (*RPL4*); *TATA box binding protein* (*TBP*).

#### Differences in gene expression across breed and sex for the controls

In order to determine the basal level gene expression differences across breed and sex only control animals (those not given a drug) were utilized in the analysis. The three-way interaction between gene, group and breed was not estimable due to some breeds not being represented across all groups and therefore could not be investigated. Significance was adjusted using the Bonferroni correction due to multiple breed/sex comparisons (n = 7). The final model utilized across target genes was:

$$y_{jklmno}\;=\;\mu +Group_{j}+\;Group\text{* }Gene_{jk}+Gene\text{*}Breed_{kl}+Gene\text{*}Sex_{km}+Animal_{n}+Animal{\left(Gene\right)}_{n\left(k\right)}+e_{jklmno},$$ (1)

where Y was the CT value, *μ* was intercept, group was the fixed effect of group_j_, group by gene was the fixed interaction of group_j_ and gene_k_, gene by breed was the fixed interaction of gene_k_ and breed_l_, gene by sex was the fixed interaction of gene_k_ and sex_m_, animal was the random effect of animal_n_, animal nested within gene was the random effect of animal_n_ within gene_k_ and e_jklmno_ was the residual.

In order to determine breed and sex expression differences for the controls the following contrasts were estimated:

Breed Contrast (TG: Target Gene; HK: Housekeeping Gene; B: Breed):

$$(TG_{B1}-\frac{1}{4}\left(HK1_{B1}+HK2_{B1}+HK3_{B1}+HK4_{B1}\right))\;-\text{ (}TG_{B2}-\frac{1}{4}\left(HK1_{B2}+HK2_{B2}+HK3_{B2}+HK4_{B2}\right)).$$

Sex Contrast (M: Male; F: Female):

$$(TG_{M}-\frac{1}{4}\left(HK1_{M}+HK2_{M}+HK3_{M}+HK4_{M}\right))\;-\text{ (}TG_{F}-\frac{1}{4}\left(HK1_{F}+HK2_{F}+HK3_{F}+HK4_{F}\right)).$$

#### Differences in gene expression across breed and sex for animals administered a drug

The number of control animals was not very large and therefore the power to detect breed and sex differences was low. Therefore, animals that were administered a drug were utilized in the analysis to determine overall gene expression differences across breed and sex and to validate the results from the previous model that only utilized the control animals. The three-way interaction between drug, group and sex was the highest order interaction investigated and it was not significant so it was removed from the model. Furthermore, the three-way interaction between gene, group and breed was not estimable due to some breeds not being represented across all groups and therefore could not be investigated. Significance was adjusted using the Bonferroni correction due to multiple breed/sex comparisons (n = 7). The final model utilized across target genes was:

$$y_{jklmno}\;=\;\mu +Group_{j}+\;Group\text{* }Gene_{jk}+Gene\text{*}Breed_{kl}+Gene\text{*}Sex_{km}+Animal_{n}+Animal{\left(Gene\right)}_{n\left(k\right)}+e_{jklmno},$$ (2)

where Y was the CT value, *μ* was intercept, group was the fixed effect of group_j_, group by gene was the fixed interaction of group_j_ and gene_k_, gene by breed was the fixed interaction of gene_k_ and breed_l_, gene by sex was the fixed interaction of gene_k_ and sex_m_, animal was the random effect of animal_n_, animal nested within gene was the random effect of animal_n_ within gene_k_ and e_jklmno_ was the residual.

In order to determine breed and sex expression differences for animals administered a drug the following contrasts were estimated:

Breed Contrast:

$$(TG_{B1}-\frac{1}{4}\left(HK1_{B1}+HK2_{B1}+HK3_{B1}+HK4_{B1}\right))\;-\;(TG_{B2}-\frac{1}{4}\left(HK1_{B2}+HK2_{B2}+HK3_{B2}+HK4_{B2}\right)).$$

Sex Contrast:

$$(TG_{M}-\frac{1}{4}\left(HK1_{M}+HK2_{M}+HK3_{M}+HK4_{M}\right))\;-\text{ (}TG_{F}-\frac{1}{4}\left(HK1_{F}+HK2_{F}+HK3_{F}+HK4_{F}\right)).$$

#### Change in gene expression due to drug administration

Controls and dosed animals were utilized in the analysis in order to characterize the change in gene expression after drug administration. The effect of treatment refers to animals that were or were not given a drug. The four-way interaction of group, treatment, sex and gene was investigated initially, was not significant, and was removed from the model. The four-way interaction between group, treatment, breed and gene was not estimable due to some breeds not being represented across all groups and therefore could not be investigated. Significance was adjusted using the Bonferroni correction due to multiple breed/sex comparisons (n = 13). The final model utilized across target genes was:

$$y_{ijklmno}\;=\;\mu +Treatment_{i}+Group_{j}+\;Group\text{* }Gene_{jk}+Treatment\text{*}Group\text{*}Gene_{ijk}+\;Treatment\text{*}Gene\text{*}Breed_{ikl}+Treatment\text{*}Gene\text{*}Sex_{ikm}+Animal_{n}+Animal{\left(Gene\right)}_{n\left(k\right)}+e_{ijklmno},$$ (3)

where Y was the CT value, *μ* was intercept, Treatment_i_ was the fixed effect of an animal receiving the drug or not, group was the fixed effect of group_j_, group by gene was the fixed interaction of group_j_ and gene_k_, treatment by group by gene was the fixed interaction of treatment_i_, group_j_ and gene_k_, treatment by gene by breed was the fixed interaction of treatment_i_, gene_k_ and breed_l_, treatment by gene by sex was the fixed interaction of treatment_i_, gene_k_ and sex_m_, animal was the random effect of animal_n_, animal nested within gene was the random effect of animal_n_ within gene_k_ and e_jklmno_ was the residual

Contrast for difference between treatment (Trt) and control (Con) to estimate change in gene expression after drug administration:

$$(TG_{Trt}-\frac{1}{4}\left(HK1_{Trt}+HK2_{Trt}+HK3_{Trt}+HK4_{Trt}\right))\;-\text{ (}TG_{Con}-\frac{1}{4}\left(HK1_{Con}+HK2_{Con}+HK3_{Con}+HK4_{Con}\right)).$$

Significant differences in gene expression change after drug administration was determined from a contrast between the respective mean difference between treatment and control for each breed or sex comparison.

Across all models contrast differences were converted to Fold Change (FC) using the following formula:

$$FC\;=\;2^{-(Contrast)}$$

## Results

### Differences in basal gene expression across breed and sex

In order to determine whether breeds or sex differed in transcript levels prior to any drug administration, the control animals were examined. The FC differences across breeds and sex based on Model 1 are outlined in Table 2. Significant (P-value < 0.007) or trending towards significant (P-value < 0.014) basal gene expression differences after Bonferonni correction were found across breeds for *SULT1A1* (Hampshire—Landrace; Hampshire—Duroc), *CYP2E1* (Yorkshire—Duroc), *CYP3A29* (Hampshire—Yorkshire) and *CYP3A22* (Hampshire-Landrace), although no sex differences were found. An alternative analysis was also constructed due to a potential lack of power to detect differences across breeds in the controls due to a small number of animals within each breed. The fold-change differences derived from Model 2 across breed and sex for animal administered flunixin meglumine and fenbendazole are outlined in S3 and S4 Tables, respectively. Three of the five across breed fold change differences for the analysis that only used the control animals were also significant (P-value < 0.007) or trending towards significant (P-value < 0.014) based on Model 2 that included only the dosed animals.

**Table 2:** Breed and sex fold change (Significance^1^) comparisons derived from the control animals across target genes.

Fold Change	*ABCB1*	*SULT1A1*	*CYP1A2*	*CYP2E1*	*CYP3A29*	*CYP3A22*
Male/Female	0.82 (0.07)	1.02 (0.91)	0.90 (0.40)	1.13 (0.33)	1.29 (0.07)	1.17 (0.28)
D/L	0.81 (0.15)	0.92 (0.68)	0.80 (0.16)	0.86 (0.42)	1.07 (0.73)	0.66 (0.04)
Y/L	0.90 (0.57)	1.53 (0.07)	1.06 (0.77)	1.59 (0.03)	1.41 (0.14)	0.75 (0.23)
H/L	0.77 (0.06)	1.82 (0.002)**	1.07 (0.68)	1.00 (0.99)	0.72 (0.08)	0.55 (0.001)**
Y/D	1.11 (0.62)	1.66 (0.05)	1.33 (0.19)	1.86 (0.009)*	1.32 (0.27)	1.14 (0.63)
H/D	0.95 (0.73)	1.98 (<0.001)**	1.34 (0.07)	1.17 (0.38)	0.68 (0.04)	0.84 (0.35)
H/Y	0.86 (0.45)	1.19 (0.46)	1.01 (0.98)	0.63 (0.03)	0.51 (0.006)**	0.74 (0.21)

^1^ The significance threshold was set at 0.007 (i.e. **) and a tendency was set at 0.014 (i.e. *) after the Bonferonni Correction.

### Change in gene expression due to drug administration

**Fig 1:**
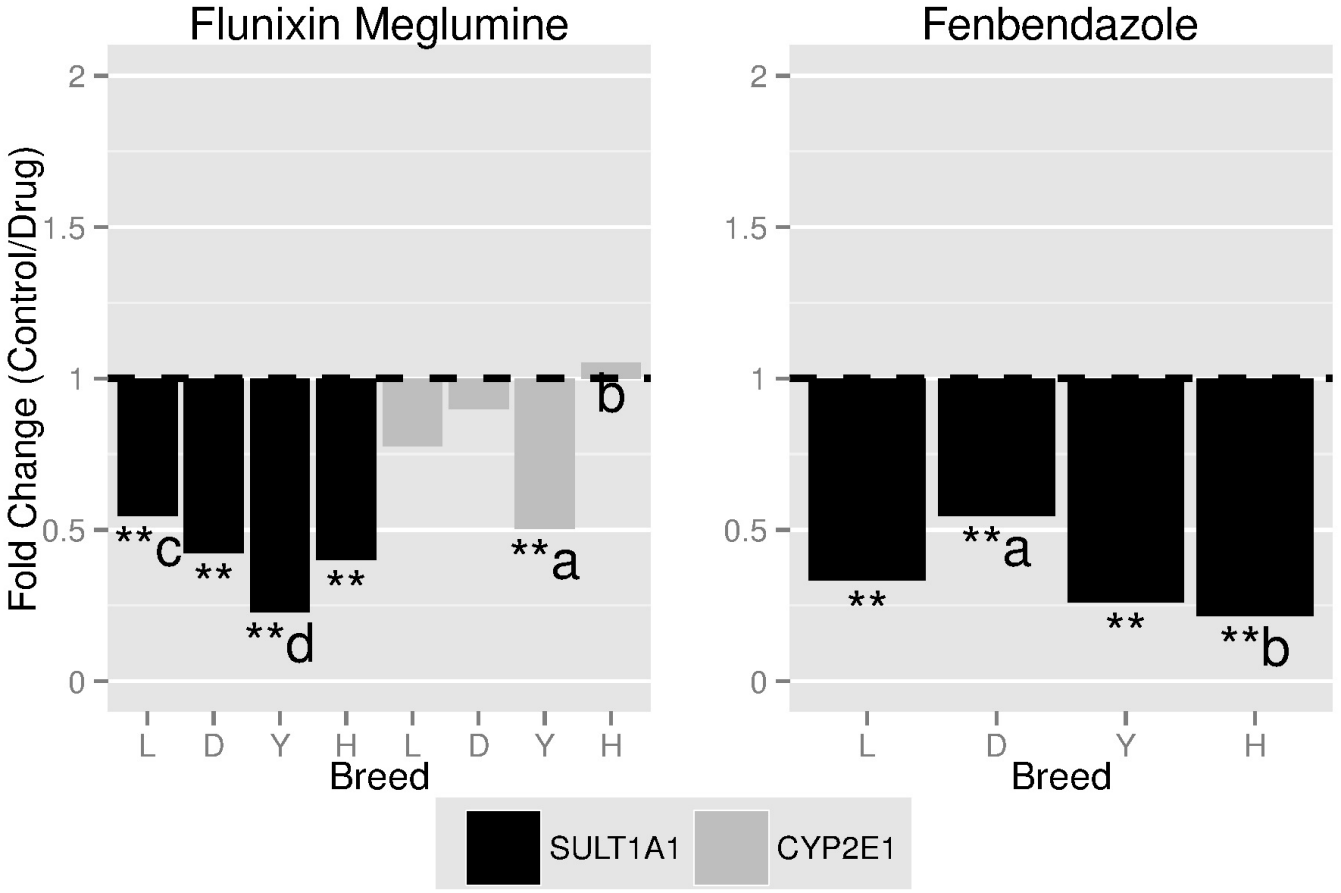
The fold-change^1,2^ between animals given a drug and the control within each breed and sex for flunixin meglumine and fenbendazole across target genes^3^. ^1^ The significance threshold was set at 0.0038 (i.e. **) and a tendency was set at 0.0076 (i.e. *) after the Bonferonni Correction. ^2^ Superscript a and b represent significant differences in fold change and c and d represent a tendency for differences in fold change. ^3^ Abbreviations: *Cytochrome P450 2E1* (*CYP2E1*); *Sulfotransferase Family*, *Cytosolic*, *1A*, *Phenol-Preferring*, *Member 1* (*SULT1A1*).

In order to determine whether breeds or sex differed in gene expression post drug administration, dosed animals were compared to the controls. The FC between dosed animals and the control within each breed and sex for flunixin meglumine and fenbendazole are outlined in Tables 3 and 4, respectively. Flunixin meglumine significantly (P-value < 0.0038) suppressed transcript levels across all four breeds and sex for *SULT1A1*. Additionally, flunixin meglumine suppressed (P-value < 0.0038) transcript levels in Landrace and Yorkshire for *ABCB1* and increased (P-value < 0.0038) transcript levels in Duroc and Yorkshire for *CYP1A2*. Lastly, as outlined in Fig 1, the fold change tended towards being significantly (P-value < 0.0076) different across breeds for *SULT1A1* (Landrace—Yorkshire) and *CYP2E1* (Yorkshire—Hampshire). Fenbendazole significantly (P-value < 0.0038) suppressed and increased transcript levels across all four breeds and sex for *SULT1A1* and *CYP1A2*, respectively. Additionally, fenbendazole significantly (P-value < 0.0038) suppressed transcript levels in Landrace for *ABCB1* and females had higher (P-value < 0.0038) transcript level after fenbendazole administration for *CYP3A29*. Lastly, the fold change, as outlined in Fig 1, was significantly (P-value < 0.0038) different across breeds for *SULT1A1* (Duroc—Hampshire) and tended towards significance (P-value < 0.0076) across sex for *CYP3A29*.

**Table 3:** The fold-change (Significance)^1^ ^,^ ^2^ between animals given a drug and the control within each breed and sex for flunixin meglumine across target genes.

	Fold Change (Drug / Control)					
	Breed (Significance)				Sex (Significance)	
Gene	Duroc	Yorkshire	Hampshire	Landrace	Male	Female
*ABCB1*	0.80 (0.06)	0.56 (0.001)**	0.94 (0.61)	0.70 (0.002)**	0.81 (0.02)	0.66 (<.001)**
*SULT1A1*	0.42 (<.001)**	0.23 (<.001)**^,c^	0.40 (<.001)**	0.55 (0.003)**^,d^	0.41 (<.001)**	0.36 (<.001)**
*CYP1A2*	1.80 (<.001)**	2.04 (0.002)**	1.41 (0.03)	1.08 (0.63)	1.70 (<.001)**	1.39 (0.01)
*CYP2E1*	0.90 (0.47)	0.50 (<.001)**^a^	1.05 (0.69)^b^	0.78 (0.07)	0.80 (0.03)	0.76 (0.01)
*CYP3A29*	1.04 (0.81)	0.78 (0.25)	1.09 (0.57)	0.81 (0.16)	0.96 (0.74)	0.88 (0.26)
*CYP3A22*	1.02 (0.92)	1.64 (0.04)	1.21 (0.28)	1.00 (0.98)	1.19 (0.18)	1.20 (0.23)

^1^ The significance threshold was set at 0.0038 (i.e. **) and a tendency was set at 0.0076 (i.e. *) after the Bonferonni Correction.

^2^ Superscript a and b represent significant differences in fold change and c and d represent a tendency for differences in fold change.

**Table 4:** The fold-change (Significance)^1^ ^,^ ^2^ between animals given a drug and the control within each breed and sex for fenbendazole across target genes.

	Fold Change (Drug / Control)					
	Breed (Significance)				Sex (Significance)	
Gene	Duroc	Yorkshire	Hampshire	Landrace	Male	Female
*ABCB1*	0.86 (0.29)	0.75 (0.16)	0.94 (0.67)	0.65 (0.003)**	0.86 (0.17)	0.73 (0.005)*
*SULT1A1*	0.55 (0.004)**^,a^	0.26 (<.001)**	0.22 (<.001)**^,b^	0.33 (<.001)**	0.33 (<.001)**	0.31 (<.001)**
*CYP1A2*	6.23 (<.001)**	3.87 (<.001)**	3.90 (<.001)**	3.45 (<.001)**	4.65 (<.001)**	3.87 (<.001)**
*CYP2E1*	1.12 (0.52)	0.68 (0.08)	0.97 (0.87)	0.92 (0.65)	0.81 (0.08)	1.02 (0.85)
*CYP3A29*	1.50 (0.03)	1.09 (0.72)	1.40 (0.08)	1.61 (0.018)	1.06 (0.66)^c^	1.81 (<.001)**^,d^
*CYP3A22*	0.83 (0.37)	1.40 (0.19)	1.44 (0.07)	1.25 (0.31)	1.01 (0.97)	1.44 (0.03)

^1^ The significance threshold was set at 0.0038 (i.e. **) and a tendency was set at 0.0076 (i.e. *) after the Bonferonni Correction.

^2^ Superscript a and b represent significant differences in fold change and c and d represent a tendency for differences in fold change.

## Discussion and Conclusions

The current analysis investigated whether there were differences between swine breeds and sex in transcript levels for genes previously shown to be involved in drug metabolism. Gene expression differences can be manifested due to baseline differences in gene expression regardless of whether a drug was given or differences in the change in transcript levels after drug administration. In regards to differences in baseline gene expression, multiple genes were found to display significant differences across breeds. In general, the Hampshire breed displayed the greatest number of pairwise breed differences across genes, including Landrace and Duroc for *SULT1A1*, Yorkshire for *CYP3A29*, and Landrace for *CYP3A22*. This is in line with previous pharmacokinetic results by Howard et al. [1], which found significant differences between Hampshire and the Landrace breeds for flunixin meglumine (clearance and volume of the distribution at steady state) and Landrace and Yorkshire for the metabolite of fenbendazole (area under the plasma concentration-time curve). No baseline gene expression differences across sex were found in the current study.

The impact of the first dose on transcript levels was assumed to be minimal due to a very small amount of drug and metabolite that remained in the blood plasma after 48 hours. To the authors’ knowledge no research has been conducted on how long gene expression is altered on the given genes when animals are administered either flunixin meglumine or fenbendazole. Furthermore, the time liver samples are collected after dosing may give rise to different gene expression results. Due to the lack of knowledge on the optimum time to collect liver samples, the results should be interpreted based on changes in transcript levels based on a liver extraction time of one hour. Future research should investigate if there is heterogeneity in transcript levels across multiple time points in order to provide a better understanding of the optimum time to collect liver samples in order to truly determine breed and sex differences. Lastly, the administration route utilized in the current study is not the approved route of drug administration for either flunixin meglumine or fenbendazole. The use of the IV route was utilized to ensure that the bioavailability of the drug was 100% and to remove inter- and intra-individual variability in drug uptake based on the approved method of oral and intramuscular administration for fenbendazole and flunixin meglumine, respectively. Peterson and Friis [24] investigated the oral bioavailability of fenbendazole in pigs and estimated it to be 27.1% and if this drug was given as a feed additive, the bioavailability may be even lower. Although the comparison between IV and oral administration on gene expression is beyond the scope of this study, one could speculate that hepatic artery delivery to the liver from IV administration may be greater than by oral administration. However, it should be noted that the feed additive is approved at 9 mg/kg BW and if bioavailability was 27%, then a pig’s liver on an oral feed additive may be exposed to more drug than the 1 mg/kg dose used in this study. The approved intramuscular method of administration for flunixin meglumine has been shown to have a bioavailability of 54–92% [26] which could be less than the IV route used in the present study and the above rational about liver exposure can also be applied.

Pharmacokinetic parameters for flunixin meglumine in swine have been well studied within a population utilizing a limited number of animals to determine the pharmacokinetic parameters [26, 29]. There is a very limited amount of research on variation across populations and no research to the author’s knowledge has been done on how flunixin meglumine impacts gene expression in swine for the genes studied. Multiple genes were found to have altered gene expression after flunixin meglumine was administered including *ABCB1*, *SULT1A1*, *CYP1A2* and *CYP2E1*. More notably, there were differences in the change in gene expression after flunixin meglumine administration across breeds for *SULT1A1* and *CYP2E1*. Therefore, the differences in pharmacokinetic parameters reported by Howard et al. [1] may be due to genetic variation that impacts drug metabolism at multiple levels including baseline levels and how it responds after drug administration.

In swine very little of fenbendazole is absorbed, but it is rapidly eliminated with a plasma half-life of approximately 2.6 hours [24]. Similar to flunixin meglumine, there has been no research to the authors’ knowledge on how fenbendazole impacts gene expression in swine for the genes studied. Multiple genes were found to have altered expression after fenbendazole was administered including *ABCB1*, *SULT1A1* and *CYP1A2*. It has been shown in rats [29] and mice [30] that fenbendazole suppresses the activity of *CYP1A1*, which is in the same family of enzymes as *CYP1A2*. The current study found an increase in *CYP1A2* expression upon fenbendazole administration. It was shown by Howard et al. [1], that the maximum metabolite concentration is reached at hour 12 for fenbendazole. Because liver samples were collected 1 hour after drug administration, the metabolite, which was shown by Murray et al. [29] to suppress *CYP1A1* activity, may not have been high enough to illicit the effect of suppressing the activity of *CYP1A2*. Moreover, there were differences in the change in gene expression after fenbendazole administration across breeds for *SULT1A1* and sex for *CYP3A29*.

*ABCB1* has been found to play a role in multi-drug resistance in humans and other species and multiple polymorphisms have been characterized but the literature is inconclusive in regards to their phenotypic effect on disease progression [31]. The *CYP1A2* has been found to be involved in the metabolism of several widely used drugs and endogenous compounds in humans and variation across ethnic groups and sex has been confirmed (see Gunes & Dahl [32], for a review). Ethnic allele frequency differences across human populations within the *CYP2E1* have also been reported [33]. Characterization of regions that are associated with skatole levels in swine has resulted in the characterization of genetic polymorphisms in the *SULT1A1* that impact sulfation activity, which is the primary method that skatole is eliminated in the liver [21]. The ability to use the *SULT1A1* polymorphisms to reduce skatole levels in order to reduce the frequency of boar taint has been suggested, although the current analysis may provide evidence that selection for reduced sulfation activity may impact the pharmacokinetic parameters of a particular drug.

In recent years, escalating costs, risks, and uncertainty associated with human drug development have posed daunting challenges to the pharmaceutical industry. This has led to initiatives to curb costs, reduce cycle times and enhance the probability of success at each stage of drug discovery and development. Furthermore, drug efficacy is generalized on a population level therefore the safety or efficacy of a molecule at a given dose in a specific population for a pre-specified disease indication is not fully known during drug discovery and development [34]. Due to these reasons, identification of suitable animal models that can be used in drug discovery will be of paramount importance in the future. With the advent of large genotyping arrays and their reduced cost, the effectiveness of the commercial pig as an animal model is greatly increased in order to better understand drug mechanisms at the molecular level. Furthermore, the swine genome has a greater degree of linkage dis-equilibrium than humans, which allows for fewer animals and markers to genotype [35]. The current study has shown that previously identified differences in pharmacokinetic parameters across breeds translated to differences at the gene expression level for flunixin meglumine and fenbendazole in the population studied. A better understanding of the molecular basis of drug action and the genetic determinants of drug response will allow for a mechanism-based approach to the discovery and development of new medications [5].

Furthermore, the within line mating structure in swine populations allows for lines/breeds to potentially have very diverse drug response phenotypes. Additional divergence among individuals can be attained in only a few selection cycles [36–37], which could potentially identify diverse drug metabolism modes of action. The efficacy of selecting individuals based on pharmacokinetic parameters in order to create divergent lines across multiple drugs will be investigated in the future by determining the heritability of the drug concentration curve and of specific pharmacokinetic parameters. Diverse drug response phenotypes can also be used to further understand drug by genotype and drug by nutrition regimen interactions, which would shed light on drug-by-drug interaction at the molecular level. Additionally, routine phenotype collection and the dense pedigree structure allows for the ability to dissect the phenotype into its genetic components and its correlation with other traits such as growth or reproductive performance traits.

The current analysis found differences across breed and sex in expression for genes previously shown to be involved in drug metabolism, including *ABCB1*, *SULT1A1*, *CYP1A2*, *CYP2E1* and *CYP3A29*. More importantly, it was found that these differences were due to inherent gene expression differences at the baseline level and differences in gene expression response after drug administration. The results provide insight into which genes are differentially expressed upon drug administration, although future research should determine the degree to which gene expression changes manifest into differences at the pharmacokinetic level. Furthermore, the differences across breeds and sex in transcript levels prior and post-drug administration confirms that breed and sex do have an effect on transcript levels for known drug metabolizing genes. Future work will determine whether genes other than the ones currently investigated are differentially expressed using RNA sequencing information and the effectiveness of clustering animals into drug efficacy groups based on pharmacokinetic parameters and gene expression data.

## Supporting Information

S1 Table Primer Information for target and housekeeping genes^1^. (DOCX)

S2 Table The number of animals by breed and sex within each group for flunixin meglumine, fenbendazole and the control used in the final analysis. (DOCX)

S3 Table Breed and sex fold change (Significance^1^) comparisons derived from animals administered Flunixin megulumine across target genes. (DOCX)

S4 Table Breed and sex fold change (Significance^1^) comparisons derived from animals administered Fenbendazole across target genes. (DOCX)

S1 File A comma separated file that includes the complete data set used in the analysis. (CSV)
