# Supplementary material for: Differential Gene Expression across Breed and Sex in Commercial Pigs Administered Fenbendazole and Flunixin Meglumine
Source: PLoS One. 2015 Sep 14;10(9):e0137830. doi: 10.1371/journal.pone.0137830 (PMC4569569; doi:10.1371/journal.pone.0137830)
Supplement: S1 Table — (DOCX) [file pone.0137830.s001.docx]

| Gene^1^ | Accession  Number | Direction | Primer Sequence 5'- 3' | cDNA  size (bp) | Primer Source |
| --- | --- | --- | --- | --- | --- |
| *ABCB1* | AY825267 | Forward | CCTGTTTGACTGCAGCATTG | 168 | Ott M, Fricker G, Bauer B. Pregnane X receptor (PXR) regulates P-glycoprotein at the blood-brain barrier: functional similarities between pig and human PXR. J Pharmacol Exp Ther. 2009; 329:141-149. |
|  |  | Reverse | GAGAGCTGCGTTCCTTTGTC |  |  |
| *SULT1A1* | AY193893.1 | Forward | ACACGTCCTTCCAGGAGAT | 162 | Seong-Jo Y, Won-Gun N, Jong-Taek Y, Kwan-Sik M. Gene Expression Analysis of Pregnant Specific Stage in the Miniature Pig Ovary  Reproductive & developmental biology, 2009 33:249-255. |
|  |  | Reverse | CAGTGATGCCTTTCCTCAT |  |  |
| *CYP1A2* | NM_001159614.1 | Forward | TTTGTGGAGACCGCCTCATC | 219 | Designed by Krista Browning using NCBI Primer Design Software (Primer 3 based) |
|  |  | Reverse | GCTGGTGCTGGAATTCTCCT |  |  |
| *CYP2E1* | AY581116 | Forward | CCAAACAGAATCCCTGCCATCAA | 183 | Kojima M, Degawa M. Serum androgen level is determined by autosomal dominant inheritance and regulates sex-related CYP genes in pigs. Biochem Biophys Res Commun. 2013 430:833-838. |
|  |  | Reverse | GGAGTCCAGTGTCGGAATTACCA |  |  |
| *CYP3A22* | NM_001195509.1 | Forward | CATTGCCCAGTATGGAGATATGC | 280 | Shang H, Guo K, Liu Y, Yang J, Wei H. Constitutive expression of CYP3A mRNA in Bama miniature pig tissues. Gene. 2013 524:261-267. |
|  |  | Reverse | AATAAATGGATCGAGGAAATCAAA |  |  |
| *CYP3A29* | NM_214423 | Forward | CTTTGATCCATTCCTTCTCTCATT | 110 | Shang H, Guo K, Liu Y, Yang J, Wei H. Constitutive expression of CYP3A mRNA in Bama miniature pig tissues. Gene. 2013 524:261-267. |
|  |  | Reverse | CTCGTAGCCAGCAAAAATAAAAATA |  |  |
| *ACTB* | XM_003357928.2 | Forward | TCGCCGACAGGATGCAGAAG | 129 | Ashwell MS, O'Nan AT, Gonda MG, Mente PL. Gene expression profiling of chondrocytes from a porcine impact injury model. Osteoarthritis Cartilage. 2008 6:936-46. |
|  |  | Reverse | AGGTGGACAGCGAGGCCAGG |  |  |
| *HPRT* | NM_001032376.2 | Forward | CAAGAGTAACTACAACCTTC | 122 | Nygard AB, Jørgensen CB, Cirera S, Fredholm M. Selection of reference genes for gene expression studies in pig tissues using SYBR green qPCR. BMC Mol Biol. 2007 8:67. |
|  |  | Reverse | GAACTCTACGATGAATCTTC |  |  |
| *RPL4* | DQ845176 | Forward | GGACTTGAATCATGTTTGTG | 91 | Nygard AB, Jørgensen CB, Cirera S, Fredholm M. Selection of reference genes for gene expression studies in pig tissues using SYBR green qPCR. BMC Mol Biol. 2007 8:67. |
|  |  | Reverse | CAGATGTTTCCAAACTCAAC |  |  |
| *TBP* | XM_005658569.1 | Forward | AACAGTTCAGTAGTTATGAGCCAGA | 160 | Nygard AB, Jørgensen CB, Cirera S, Fredholm M. Selection of reference genes for gene expression studies in pig tissues using SYBR green qPCR. BMC Mol Biol. 2007 8:67. |
|  |  | Reverse | AGATGTTCTCAAACGCTTCG |  |  |

^1^ Abbreviations: *ATP-Binding Cassette Sub-Family B* (*ABCB1*); *Sulfotransferase Family, Cytosolic, 1A, Phenol-Preferring, Member 1* (*SULT1A1*); *Cytochrome P450 1A2* (*CYP1A2*); *Cytochrome P450 1E2* (*CYP2E1*); *Sulfotransferase Family, Cytochrome P450 3A22* (*CYP3A22*); *Cytochrome P450 3A29* (CYP3A29); *Beta-actin* (*ACTB*); *hypoxanthine phosphoribosyltransferase 1* (*HPRT1*); *ribosomal protein L4* (*RPL4*); *TATA box binding protein* (*TBP*).
