# Supplementary material for: Differential Gene Expression across Breed and Sex in Commercial Pigs Administered Fenbendazole and Flunixin Meglumine
Source: PLoS One. 2015 Sep 14;10(9):e0137830. doi: 10.1371/journal.pone.0137830 (PMC4569569; doi:10.1371/journal.pone.0137830)
Supplement: S2 Table — (DOCX) [file pone.0137830.s002.docx]

| Treatment | Gene | Breed (Group) | | | | Sex (Group) | |
| --- | --- | --- | --- | --- | --- | --- | --- |
|  |  | Duroc | Yorkshire | Hampshire | Landrace | Female | Male |
| flunixin meglumine | *ABCB1* | 23 (4-10-4-1-4) | 13 (3-0-2-1-7) | 17 (4-4-3-2-4) | 14 (3-2-2-5-2) | 32 (7-8-3-4-10) | 35 (7-8-8-5-7) |
|  | *SULT1A1* | 22 (4-9-4-1-4) | 14 (3-0-2-2-7) | 19 (4-4-3-4-4) | 15 (4-2-2-5-2) | 32 (7-8-3-4-10) | 38 (8-7-8-8-7) |
|  | *CYP1A2* | 23 (4-10-4-1-4) | 13 (3-0-2-1-7) | 19 (4-4-3-4-4) | 15 (4-2-2-5-2) | 32 (7-8-3-4-10) | 38 (8-8-7-8-7) |
|  | *CYP2E1* | 19 (4-6-4-1-4) | 12 (3-0-1-1-7) | 18 (4-4-3-3-4) | 14 (3-2-2-5-2) | 29 (6-6-3-4-10) | 34 (8-6-7-6-7) |
|  | *CYP3A22* | 21 (4-9-3-1-4) | 14 (3-0-2-2-7) | 19 (4-4-3-4-4) | 14 (4-2-1-5-2) | 32 (7-8-3-4-10) | 36 (8-7-6-8-7) |
|  | *CYP3A29* | 22 (3-10-4-1-4) | 13 (3-0-2-1-7) | 19 (4-4-3-4-4) | 15 (4-2-2-5-2) | 31 (6-8-3-4-10) | 38 (8-8-8-7-7) |
|  | *ACTB* | 22 (4-10-3-1-4) | 14 (3-0-2-2-7) | 19 (4-4-3-4-4) | 15 (4-2-2-5-2) | 32 (7-8-3-4-10) | 38 (8-8-7-8-7) |
|  | *HPRT* | 23 (4-10-4-1-4) | 13 (3-0-1-2-7) | 19 (4-4-3-4-4) | 15 (4-2-2-5-2) | 32 (7-8-3-4-10) | 38 (8-8-7-8-7) |
|  | *RPL4* | 22 (4-9-4-1-4) | 13 (3-0-2-2-6) | 19 (4-4-3-4-4) | 15 (4-2-2-5-2) | 30 (7-7-3-4-9) | 39 (8-8-8-8-7) |
|  | *TBP* | 20 (3-8-4-1-4) | 13 (3-0-1-2-7) | 19 (4-4-3-4-4) | 15 (4-2-2-5-2) | 30 (6-7-3-4-10) | 37 (8-7-7-8-7) |
| fenbendazole | *ABCB1* | 16 (4-4-3-1-4) | 14 (4-0-4-2-4) | 14 (4-0-3-4-3) | 11 (4-1-2-3-1) | 26 (9-2-4-5-6) | 29 (7-3-8-5-6) |
|  | *SULT1A1* | 17 (4-4-4-1-4) | 15 (4-0-4-3-4) | 16 (4-0-3-6-3) | 11 (4-1-2-3-1) | 27 (9-2-4-6-6) | 32 (7-3-9-7-6) |
|  | *CYP1A2* | 16 (3-4-4-1-4) | 15 (4-0-4-3-4) | 16 (4-0-3-6-3) | 10 (2-1-2-4-1) | 27 (9-2-4-6-6) | 30 (4-3-9-8-6) |
|  | *CYP2E1* | 16 (4-3-4-1-4) | 15 (4-0-4-3-4) | 15 (4-0-2-6-3) | 10 (4-1-1-3-1) | 26 (9-1-4-6-6) | 30 (7-3-7-7-6) |
|  | *CYP3A22* | 15 (4-3-3-1-4) | 14 (4-0-4-2-4) | 14 (4-0-2-5-3) | 9 (4-1-0-3-1) | 23 (9-1-3-4-6) | 29 (7-3-6-7-6) |
|  | *CYP3A29* | 17 (4-4-4-1-4) | 13 (4-0-4-1-4) | 16 (4-0-3-6-3) | 10 (4-1-1-3-1) | 25 (9-2-3-5-6) | 31 (7-3-9-6-6) |
|  | *ACTB* | 17 (4-4-4-1-4) | 14 (4-0-4-2-4) | 16 (4-0-3-6-3) | 11 (4-1-2-3-1) | 26 (9-2-4-5-6 | 32 (7-3-9-6-7) |
|  | *HPRT* | 16 (4-4-4-1-3) | 14 (4-0-3-3-4) | 15 (4-0-2-6-3) | 11 (4-1-2-2-2) | 25 (9-2-3-6-5) | 31 (7-3-8-7-6) |
|  | *RPL4* | 17 (4-4-4-1-4) | 15 (4-0-4-3-4) | 16 (4-0-3-6-3) | 10 (4-1-2-3-0) | 26 (9-2-4-6-5) | 32 (7-3-9-7-6) |
|  | *TBP* | 16 (4-3-4-1-4) | 15 (4-0-4-3-4) | 16 (4-0-3-6-3) | 10 (4-1-2-3-0) | 25 (9-1-4-6-5) | 32 (7-3-9-7-6) |
| control | *ABCB1* | 10 (2-4-1-1-2) | 4 (1-0-1-2-0) | 11 (2-3-1-3-2) | 12 (2-2-3-3-2) | 19 (4-4-2-6-3) | 18 (3-5-4-3-3) |
|  | *SULT1A1* | 10 (2-4-1-1-2) | 6 (2-0-1-3-0) | 11 (2-2-1-4-2) | 10 (2-2-2-3-1) | 18 (4-4-1-7-2) | 19 (4-4-4-4-3) |
|  | *CYP1A2* | 10 (2-4-1-1-2) | 5 (1-0-1-3-0) | 11 (2-3-1-3-2) | 11 (2-2-2-3-2) | 18 (4-4-1-6-3) | 19 (3-5-4-4-3) |
|  | *CYP2E1* | 9 (2-4-0-1-2) | 5 (1-0-1-3-0) | 12 (2-3-1-4-2) | 11 (2-1-3-3-2) | 19 (4-3-2-7-3) | 18 (3-5-3-4-3) |
|  | *CYP3A22* | 9 (2-4-0-1-2) | 5 (1-0-1-3-0) | 11 (2-3-0-4-2) | 10 (2-2-1-3-2) | 18 (4-4-0-7-3) | 17 (3-5-2-4-3) |
|  | *CYP3A29* | 10 (2-4-1-1-2) | 5 (1-0-1-3-0) | 11 (2-3-0-4-2) | 12 (2-2-3-3-2) | 20 (4-4-2-7-3) | 18 (3-5-3-4-3) |
|  | *ACTB* | 10 (2-4-1-1-2) | 5 (1-0-1-3-0) | 12 (2-3-1-4-2) | 12 (2-2-3-3-2) | 20 (4-4-2-7-3) | 19 (3-5-4-4-3) |
|  | *HPRT* | 10 (2-4-1-1-2) | 4 (1-0-1-2-0) | 11 (2-3-0-4-2) | 11 (2-2-3-3-1) | 18 (4-4-2-6-2) | 18 (3-5-3-4-3) |
|  | *RPL4* | 9 (2-4-1-1-1) | 5 (1-0-1-3-0) | 12 (2-3-1-4-2) | 12 (2-2-3-3-2) | 19 (4-4-2-7-2) | 19 (3-5-4-4-3) |
|  | *TBP* | 10 (2-4-1-1-2) | 6 (2-0-1-3-0) | 12 (2-3-1-4-2) | 12 (2-2-3-3-2) | 20 (4-4-2-7-3) | 20 (4-5-4-4-3) |
