# Supplementary material for: Differential Gene Expression across Breed and Sex in Commercial Pigs Administered Fenbendazole and Flunixin Meglumine
Source: PLoS One. 2015 Sep 14;10(9):e0137830. doi: 10.1371/journal.pone.0137830 (PMC4569569; doi:10.1371/journal.pone.0137830)
Supplement: S3 Table — (DOCX) [file pone.0137830.s003.docx]

| Fold Change | *ABCB1* | *SULT1A1* | *CYP1A2* | *CYP2E1* | *CYP3A29* | *CYP3A22* |
| --- | --- | --- | --- | --- | --- | --- |
| Male/Female | 1.00 (0.95) | 1.16 (0.24) | 1.10 (0.39) | 1.12 (0.03) | 1.42 (<0.001)^1^** | 1.17 (0.16) |
| D/L | 0.93 (0.43) | 0.71 (0.06) | 1.32 (0.06) | 1.00 (0.97) | 1.36 (0.02) | 0.67 (0.013)* |
| Y/L | 0.71 (0.003)** | 0.64 (0.02) | 2.00 (<0.001)** | 1.04 (0.80) | 1.36 (0.03) | 1.22 (0.25) |
| H/L | 1.04 (0.70) | 1.34 (0.10) | 1.40 (0.03) | 1.36 (0.009)* | 0.97 (0.77) | 0.66 (0.01)* |
| Y/D | 0.77 (0.015)* | 0.90 (0.55) | 1.51 (0.014)* | 1.04 (0.76) | 1.00 (0.99) | 1.85 (<0.001)** |
| H/D | 1.12 (0.19) | 1.87 (0.001)** | 1.06 (0.71) | 1.37 (0.004)** | 0.71 (0.003)** | 1.00 (0.98) |
| H/Y | 1.46 (<0.001)** | 2.09 (<0.001)** | 0.70 (0.03) | 1.31 (0.03) | 0.71 (0.01)* | 0.54 (<0.001)** |

^1^ The significance threshold was set at 0.007 (i.e. **) and a tendency was set at 0.014 (i.e. *) after the Bonferonni Correction.
