# Supplementary material for: Differential Gene Expression across Breed and Sex in Commercial Pigs Administered Fenbendazole and Flunixin Meglumine
Source: PLoS One. 2015 Sep 14;10(9):e0137830. doi: 10.1371/journal.pone.0137830 (PMC4569569; doi:10.1371/journal.pone.0137830)
Supplement: S4 Table — (DOCX) [file pone.0137830.s004.docx]

| Fold Change | ABCB1 | SULT1A1 | CYP1A2 | CYP2E1 | CYP3A29 | CYP3A22 |
| --- | --- | --- | --- | --- | --- | --- |
| Male/Female | 0.97(0.75) | 1.10(0.50) | 1.09 (0.56) | 0.89 (0.29) | 0.76 (0.04) | 0.82 (0.15) |
| D/L | 1.07(0.62) | 1.51(0.06) | 1.44 (0.10) | 1.05 (0.77) | 0.99 (0.97) | 0.44 (<0.001)^1^** |
| Y/L | 1.03(0.82) | 1.19(0.43) | 1.19 (0.43) | 1.17 (0.35) | 0.96 (0.82) | 0.84 (0.41) |
| H/L | 1.11(0.44) | 1.18(0.44) | 1.20 (0.39) | 1.06 (0.74) | 0.63 (0.02) | 0.64 (0.03) |
| Y/D | 0.96(0.77) | 0.79(0.24) | 0.83 (0.34) | 1.12 (0.46) | 0.96 (0.83) | 1.90 (<0.001)** |
| H/D | 1.04(0.78) | 0.78(0.23) | 0.83 (0.36) | 1.01 (0.96) | 0.63 (0.01)* | 1.45 (0.05) |
| H/Y | 1.08(0.56) | 0.99(0.98) | 1.01 (0.97) | 0.90 (0.50) | 0.66 (0.02) | 0.76 (0.15) |

^1^ The significance threshold was set at 0.007 (i.e. **) and a tendency was set at 0.014 (i.e. *) after the Bonferonni Correction.
